# Functions of CNKSR2 and Its Association with Neurodevelopmental Disorders

**DOI:** 10.3390/cells11020303

**Published:** 2022-01-17

**Authors:** Hidenori Ito, Koh-ichi Nagata

**Affiliations:** 1 Department of Molecular Neurobiology, Institute for Developmental Research, Aichi Developmental Disability Center, 713-8 Kamiya, Kasugai 480-0392, Japan; knagata@inst-hsc.jp; 2 Department of Neurochemistry, Nagoya University Graduate School of Medicine, 65 Tsurumai-cho, Showa-ku, Nagoya 466-8550, Japan

**Keywords:** CNKSR2, epilepsy, neurodevelopmental disorder, scaffold protein, disease

## Abstract

The Connector Enhancer of Kinase Suppressor of Ras-2 (CNKSR2), also known as CNK2 or MAGUIN, is a scaffolding molecule that contains functional protein binding domains: Sterile Alpha Motif (SAM) domain, Conserved Region in CNK (CRIC) domain, PSD-95/Dlg-A/ZO-1 (PDZ) domain, Pleckstrin Homology (PH) domain, and C-terminal PDZ binding motif. CNKSR2 interacts with different molecules, including RAF1, ARHGAP39, and CYTH2, and regulates the Mitogen-Activated Protein Kinase (MAPK) cascade and small GTPase signaling. CNKSR2 has been reported to control the development of dendrite and dendritic spines in primary neurons. CNKSR2 is encoded by the *CNKSR2* gene located in the X chromosome. *CNKSR2* is now considered as a causative gene of the Houge type of X-linked syndromic mental retardation (MRXHG), an X-linked Intellectual Disability (XLID) that exhibits delayed development, intellectual disability, early-onset seizures, language delay, attention deficit, and hyperactivity. In this review, we summarized molecular features, neuronal function, and neurodevelopmental disorder-related variations of *CNKSR2*.

## 1. Introduction

Neurodevelopmental disorders are a heterogeneous group of disorders, including Intellectual Disability (ID), autism spectrum disorder, Attention Deficit Hyperactivity Disorder (ADHD), and epilepsy [1,2]. The prevalence of ID is often estimated at 1% [3], while a more recent report showed that the prevalence might be lower than 1% [4,5]. The occurrence of ID is higher in males than in females, probably because of X-linked recessive inheritance. Since the discovery of *FMR1* variants in patients with Fragile X Syndrome [6], over 140 genes have been associated with X-linked Intellectual Disability (XLID) [7]. The Connector Enhancer of Kinase Suppressor of Ras 2 (CNKSR2) is a synaptic scaffold protein encoded by the *CNKSR2* gene (NCBI Reference Sequence: NM_014927) located on the X chromosome (Xp22.12). The variant of *CNKSR2* in XLID was found in 2012 [8], and reports describing the implication of CNKSR2 in neurodevelopmental disorders are increasing now. In this review, we firstly summarize the molecular characteristics and biological functions of CNKSR2 and then we describe the involvement of *CNKSR2* in neurodevelopmental disorders.

## 2. Molecular Characteristics of CNKSR2

**Figure 1:**
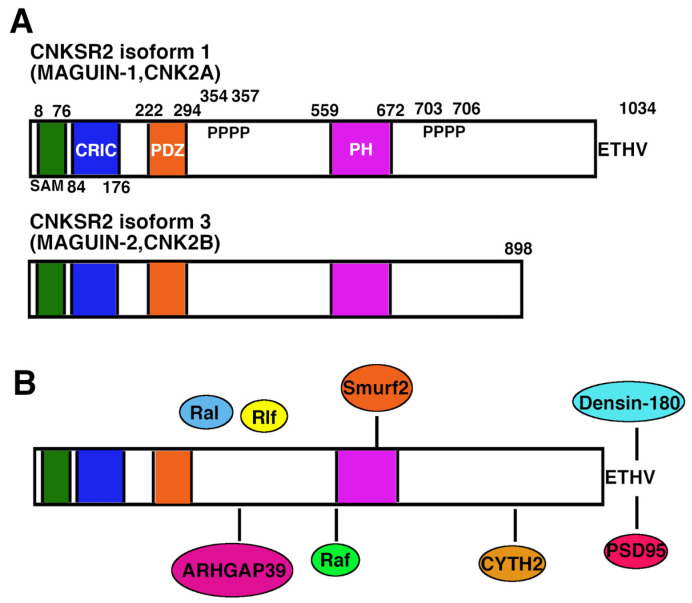
Molecular structure and interacting molecules of CNKSR2. (**A**) Structure of CNKSR2. SAM, the sterile alpha motif; CRIC, the conserved region in CNKSR2; PDZ, PDZ domain; PH, pleckstrin homology domain. Numbers indicate amino acid positions. (**B**) Interacting partners for CNKSR2. Binding regions of CNKSR2 and interacting partners are connected with solid lines. The full-length molecule of CNKSR2 may be required for binding with Rlf, because neither *N*- nor C-terminal fragment interact. The region in CNKSR2 responsible for binding to Ral has not been obtained because of the weak interaction.

CNKSR2, also known as CNK2 or MAGUIN, was first identified as a binding partner for Postsynaptic Density (PSD)-95 and Synaptic Scaffolding Molecule (S-SCAM) [9]. CNKSR2 has a molecular structure similar to CNK found in *Drosophila*, which regulates the eyes and wing development by regulating Ras/Mitogen-activated Protein Kinase (MAPK) signaling [10]. Two isoforms named CNK2A and CNK2B, now assigned as CNKSR2 isoform 1 and isoform 3, respectively, have been identified in humans [11], which have amino acid sequences nearly identical to rat MAGUIN-1 and MAGUIN-2, respectively [7]. CNKSR2 isoform 1 consists of 1034 aa involving one Sterile Alpha Motif (SAM) domain, one Conserved Region in CNKSR (CRIC) domain, one PSD-95/Dlg-A/ZO-1 (PDZ) domain, one Pleckstrin Homology (PH) domain, and one C-terminal PDZ binding motif [9,11] (Figure 1A). The SAM domain is an evolutionarily conserved protein binding domain found in molecules involved in numerous developmental processes and signal transduction pathways of diverse organisms, including fungi, protozoa, and mammals [12]. The PDZ domain is one of the most common protein interaction domains, and many PDZ domain-containing proteins participate in the signaling machinery at neuronal synapses [13]. The CRIC domain is a leucine-enriched domain that functions as a protein-protein interacting domain [14,15]. The PH domain is a small modular domain found in a wide variety of proteins that can bind lipid phosphatidylinositol and proteins, including protein kinase C and beta/gamma subunit of heterotrimeric G protein [16]. CNKSR2 isoform 3 is a truncated form of isoform1 consisting of 898 aa lacking a C- terminal PDZ binding motif [9,11] (Figure 1A). Northern blotting revealed the expression of *CNKSR2* mRNA only in the brain [9]. Biochemical fractionation of rat brains revealed the existence of CNKSR2 in the synaptic plasma membrane and post synaptic density fractions [9]. CNKSR2 colocalized with NMDAR1 and PSD95 in neurites of primary cultured neurons [9,17,18].

## 3. Interaction of CNKSR2 with Cellular Signaling Molecules

CNKSR2 is recognized as a multidomain scaffold molecule, and several interactors have been reported (summarized in Figure 1B).

### 3.1. CNKSR2 and Kinase Signaling Pathway

The functional association of CNKSR2 in Extracellular Signal-Regulated Kinase (ERK) signaling has been reported [11,19]. Treatment with pervanadate, an inhibitor of tyrosine phosphatases, increased the phosphorylation of transiently expressed CNKSR2 in HEK293 cells [11]. The constitutively active RasV12-induced phosphorylation of CNKSR2 was suppressed by mitogen-activated protein kinase kinase (MEK) inhibitor but not by phosphatidylinositol 3-kinase (PI3K) inhibitor [11]. These results suggest that CNKSR2 is phosphorylated downstream by ERK kinase signaling.

Regulation of ERK activation by CNKSR2 has also been reported. CNKSR2 can form a complex with RAF1/c-Raf [11,19], a member of evolutionarily conserved cytosolic serine/threonine kinase RAF family. RAFs play pivotal roles in activating ERK and regulating various cellular processes such as proliferation, migration, differentiation, and survival [20]. The PH domain-containing region of CNKSR2 was responsible for the interaction with kinase domain [11,19] and regulatory domain [11] of RAF1. Overexpression of CNKSR2 suppressed the RasV12-stimulated ERK activation [11].

In PC12 cells, the knockdown of CNKSR2 resulted in the inhibition of NGF-induced neurite outgrowth [21]. Further analysis revealed the requirement of CNKSR2 for NGF but not EGF-induced activation of ERK [21]. Expression of the *N*-terminal half of CNKSR2 (1–468) significantly inhibited NGF-induced neurite outgrowth of PC12 cells without affecting ERK activation [21]. These results suggest that CNKSR2 regulates the differentiation of PC12 cells through ERK-independent pathways.

### 3.2. CNKSR2 and Small GTPase Signaling

CNKSR2 interacts with proteins in Ral small GTPase pathways [11]. CNK2B (CNKSR2 isoform 3) is bound with Ral stimulator Rlf much stronger than Ral while CNK2B weakly interacted with Ral in the presence of Ral stimulator Ras V12 [11]. Further analysis revealed that CNK2A (CNKSR2 isoform 1) and CNK2B equally interact with Rlf [11]. Expression of Rlf caused an increase of CNK2B in the membrane fraction, but overexpression of CNK2A did not affect the activation of Rlf-mediated Ral activation [11]. These results suggest that CNKSR2 may function as an adaptor protein or regulator of multiple Ras signaling pathways. However, precise roles have yet to be clarified.

Mass spectrometry analysis of endogenous CNKSR2 complexes isolated from NG108 cells revealed the interaction with the Rac GTPase signaling molecules, Vilse/ARHGAP39, PAK3, PAK4, ARHGEF6, ARHGEF7, and CYTH2/cytohesin-2/ARNO [18]. Vilse/ARHGAP39 is the GTPase Activating Protein (GAP) for Rac, which acts downstream of Robo to regulate Rac-mediated midline repulsion in *Drosophila* [22,23]. In mammals, Vilse/ARHGAP39 regulates dendritic architecture and synaptic plasticity [24]. Mapping analysis revealed that proline-rich motif at amino acid positions 354–357 of CNKSR2 binds with two WW domains containing *N*-terminal region of Vilse/ARHGAP39 [18]. CNKSR2 did not alter the GAP activity of Vilse/ARHGAP39 but controlled its localization at the plasma membrane [18]. SiRNA-mediated knockdown of *CNKSR2* impaired the NGF-induced neurite growth and GTP loading (activation) of Rac in NG108 cells [18]. In primary cultured hippocampal neurons, endogenous CNKSR2 and Vilse/ARHGAP39 colocalized with the excitatory synaptic marker PSD95 [18]. The knockdown of *CNKSR2* caused a reduction in the number of mature spines, whereas the number of protrusions was increased [18]. These effects were restored by the expression of an RNAi-resistant version of CNKSR2 although CNKSR2 mutants of the proline-rich motif, which lack binding to Vilse/ARHGAP39, did not [18]. These results suggest significant roles of CNKSR2-Vilse/ARHGAP39 complex in spine formation.

Recently, we clarified the participation of CNKSR2 in the development of the hippocampus [25]. We discovered that co-expression of CNKSR2 with CYTH2 or ARHGAP39 but not ARHGEF7 increased expression of CNKSR2. CYTH2 is a guanine nucleotide exchange factor for the ARF small GTPase [26] that regulates the neurite formation of primary hippocampal neurons [27,28,29]. We showed that CYTH2 binding prevents proteasomal degradation of CNKSR2. To reveal the function of the CNKSR2-CYTH2 complex, we employed the in vivo electroporation-mediated method using neonatal mouse [30]. We found that the knockdown of CNKSR2 or CYTH2 caused the mislocalization of neonatally-born dentate granule cells. The mislocalized cells exhibited features of immature dentate granule cells.

### 3.3. Interaction of CNKSR2 with Synaptic Molecules

As described, CNKSR2 was identified as a binding protein for neuronal membrane-associated guanylate kinase, PSD95 and S-SCAM/MAGI-2 [9]. S-SCAM/MAGI-2 is also named as Atrophin-1-Interacting Protein 1 (AIP-1) or Activin Receptor-Interacting Protein 1 (ARIPI1) [31,32]. S-SCAM/MAGI-2 is expressed mainly in the brain and interacts with Synapse-Associated Protein-Associated Protein (SAPAP)/Guanylate Kinase Associated Protein (GKAP), NMDA receptor, and neuroligin [33]. Gene variations of *S-SCAM/MAGI-2* are associated with schizophrenia [34,35] and infantile spasm [36]. MAGUIN-1 (CNKSR2 isoform 1) but not MAGUIN-2 (CNKSR2 isoform 3) formed a complex with S-SCAM/MAGI-2 [9]. The fourth and fifth PDZ domains can interact with the C-terminus PDZ binding motif of MAGUIN-1 [9]. These results may indicate the significance of CNKSR2 in the synaptic organization.

Densin-180 was reported to form a complex with CNKSR2 [37]. Densin-180 is a brain-enriched protein that contains 16 Leucine-Rich Repeats (LRRs) and a PDZ domain [38]. The C-terminus of CNKSR2 interacts with the PDZ domain of Densin-180, and these molecules colocalize at the dendritic spine of primary cultured hippocampal neurons [37]. However, the physiological role of the interaction is unclear to date.

TRAF2- and NCK-Interacting Kinase (TNIK) is a member of Ste20 like kinase and specifically activates the c-Jun *N*-terminal kinase pathway [39]. TNIK regulates protein complexes in PSD, and gene knockout in mice exhibit the impairment of the cognitive function of hyperactivity [40]. Gene variants of *TNIK* have been found in patients with ID [41]. Recently, the interaction between CNKSR2 and TNIK was reported [42]. CNKSR2 and TNIK colocalized at dendritic spines of primary cultured neuron and the expression of CNKSR2 lacking PH domain reduced the dendritic the localization of TNIK in primary cultured neuron [42]. The function of CNKSR2 to regulate localization of TNIK and other synaptic molecules may be required for the proper synaptic development in neurons.

### 3.4. Interaction of CNKSR2 to Other Signaling Molecules

CNKSR2 interacts with tumor suppressor DAL-1/band 4.1B [21]. DAL-1/band 4.1B is a member of the Protein 4.1 superfamily, characterized by the presence of a FERM (Four.1 protein, Ezrin, Radixin, Moesin) domain at the *N*-terminus and is known as a tumor suppressor and a stabilizer of spectrin/actin cytoskeleton [43,44,45]. Mapping analysis revealed that the C-terminal half of CNKSR2 interacts with DAL-1/band 4.1 B [21]. The knockdown of DAL-1/band 4.1 B suppressed NGF-induced neurite outgrowth of PC12 cells without inhibiting ERK activity [21]. As described, CNKSR2 also controls NGF-induced neurite extension of PC12 cells in an ERK activation-independent manner [21]. These results indicate that CNKSR2 and DAL-1/band 4.1 B co-operatively regulate NGF-induced cytoskeletal rearrangement required for neurite extension.

The interaction between CNKSR2 and Smurf2 was reported [46]. Smurf2 (Smad ubiquitin regulatory factor 2) is a member of the Nedd4 family of HECT ubiquitin ligases that negatively regulates TGF-ß signaling [47]. The WW2 domain of Smurf2 was responsible for the interaction with the ‘SPPPPY’ motif at aa702-707 in CNKSR2 [46]. Smurf2 positively regulated the CNKSR2 expression level at the post-translational level, even though Smuf2 is a molecule that facilitates the ubiquitination of protein. Smuf2 knockdown downregulated the proliferation of breast cancer cells by modulating the PI3K-PTEN-AKT-FoxO3a pathway via CNKSR2 [46].

## 4. CNKSR2 and Neurodevelopmental Disorders

**Figure 2:**
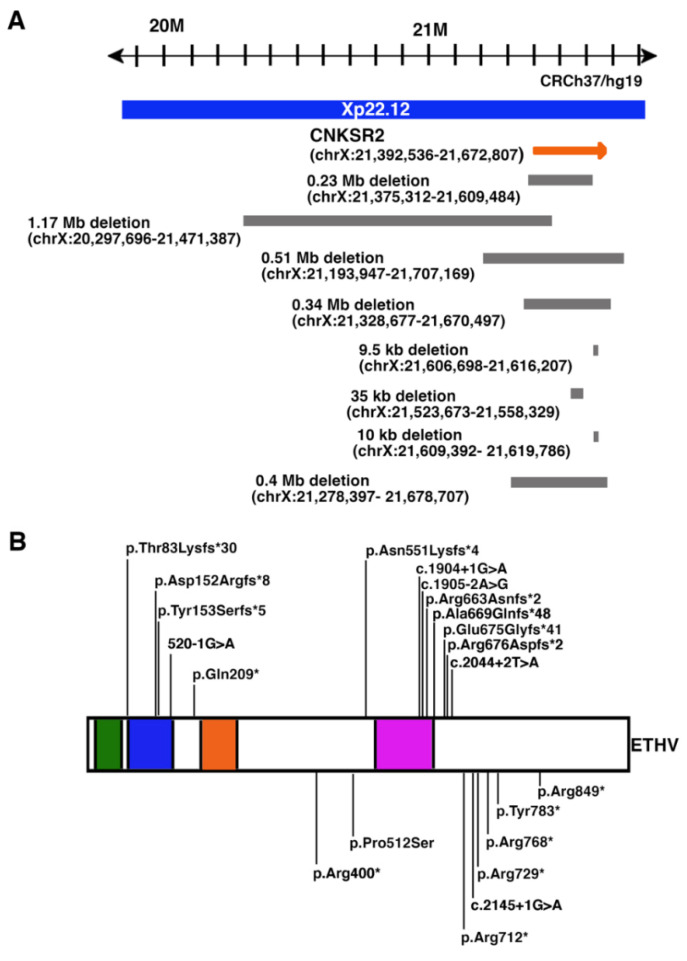
Schematic representation of human CNKSR2 variants identified in patients with neurodevelopmental disorder. (**A**) Genomic map of chromosome Xp22.12 with extent of deletions (gray bars). (**B**) Illustration of human CNKSR2 including the positions and the predicted effect of the variants.

XLID is a generalized neurodevelopmental disorder caused by gene defects on the X chromosome. XLID has been estimated to account for about 10% of the cause in males with ID and much lower cases in females [48,49,50,51,52]. *CNKSR2* is now considered as a causative gene of the Houge type of X-linked syndromic mental retardation (MRXHG), an XLID recorded in OMIN in 2017 (#301008). MRXSHG is characterized by delayed development, ID, early-onset seizures, and language delay. Serious attention deficit and hyperactivity have frequently been observed [53]. Multiple types of variations, including deletion [8,54,55,56,57], frame shift [54,57,58], splicing [57,59], and nonsense variations [53,57,60,61,62] have been reported in patients suffering from MRXSHG (Figure 2 and Table 1). Severely affected patients were all hemizygous males and heterozygous female carriers of variants that exhibited moderate to mild phenotype or unaffected (Table 1), probably because of the production of CNKSR2 from the normal allele. *CNKSR2* is selectively expressed in the human brain, and the expression is widely observed in various brain regions, including the hippocampus, amygdala, and cerebellum [63,64]. This broad expression of in the brain may cause different symptoms observed in many patients (Table 1). 

### 4.1. Deletion of Xp22.12 Involving the CNKSR2 Gene

Houge et al. firstly reported the case of a non-dysmorphic 5-years-old boy with developmental delay, ADHD, well-controlled epilepsy, and microcephaly [8]. Copy number analysis revealed a 0.23-Mb deletion of Xp22.12 (21,375,312–21,609,484), removing the initial 15 of 21 exons of the gene. The deletion was inherited from his mother but she did not report a learning difficulty.

The 1.17-Mb deletion (Xp22.12; 20,297,696–21,471,387) and 0.51-Mb deletion (Xp22.12; 21,193,947–21,707,169) were found in male patients with ID, attention problems, and abrupt lifelong language loss following a brief early childhood epilepsy with Continuous Spikes and Waves during Slow Sleep (CSWS) [54]. The 0.34-Mb deletion (Xp22.12; 21,328,677–21,670,497) [55] and the 9.5-kb deletion of (Xp22.12; 21,606,698–21,616,207) [56] have been reported in male patients with medically intractable focal seizures, developmental delay, ID, and language delay.

All mothers of probands described above had the deletion of Xp22.12; 21,375,312–21,609,484 [8], Xp22.12; 20,297,696–21,471,387 [54], and Xp22.12; 21,606,698–21,616,207 (described as the heterozygous deletion in [56]), but exhibited no or very mild symptoms of neurodevelopmental disorders. These mild symptoms could be explained by the heterozygosity of females. However, a female patient with a 35-kb deletion involving the gene (Xp22.12; 21,523,673–21,558,329) exhibited symptoms of neurodevelopmental disorders, including neurodevelopment regression, language defects, and hyperactivity after the onset of seizures [58], indicating additional mechanisms may contribute to the pathogenesis of the disorder. In the same study, the authors reported a male with the deletion of 10-kb on Xp22.12 (21,609,392–21,619,786), including the gene. He presented attention deficit, moderate neurodevelopmental delay, hypotonia, epilepsy, and severe language delay. In another case, the deletion on Xp22.12 (21,278,397–21,678,707) was found in a male [57]. His electroencephalogram (EEG) demonstrated seizures during both day and night. Brain abnormality was reported, but details were unknown. He also had language delay, hyperactivity, and autism.

### 4.2. Nonsense Variants of CNKSR2

Analyses of patients with the Epilepsy-Aphasia Spectrum (EAS) revealed c.2134C > T; p.(Arg712*) (Reference sequence, NM_014927) (The authors described ’c.2314C > T; p.(Arg712*)’ in the paper [53]; but we indicated the nucleic acid number in the reference sequence), c.2185C > T; p.(Arg729*) [60] and c.2349T > G; p.(Tyr783*) [57] nonsense mutations in the *CNKSR2* gene. EAS is a spectrum disorder from the severe end of Landau-Kleffner syndrome [65] and CSWS to the mild end of Childhood Epilepsy with Centrotemporal Spikes (CECTS) [66]. The male proband described by Damiano et al. had severe CSWS, developmental and language delay, and ADHD before the seizure onset [53]. The proband’s brother exhibited similar symptoms, although he was affected less severely than the proband. The proband’s sister with the heterozygous variant showed mild motor and language delay, and she was diagnosed with CECTS [53]. Her mother who also had the heterozygous variant had febrile seizures while she did not have language and intellectual impairment [53]. Another female who had the heterozygous variant of c.2304G > A; p.(Arg768*) in *CNKSR2* showed mild ID and seizures [61]. She had a delay of motor development, mild facial dysmorphism, and abnormalities of the extremities.

Recently, males who had c.625C > T; p.(Gln209*) [62], c.2545C > T; p.(Arg849*) [57] and c.1198C > T, p.(Arg400*) [57] were reported. These patients were featured with ID, language defect, epilepsy, and hyperactivity.

### 4.3. Frameshift Variants of CNKSR2

A frameshift-premature termination mutation in the *CNKSR2* (g.21,458,832_3insA; p.(D152Rfs*8)) was reported in three males who had language defect, childhood seizures, and ADHD [54]. In another unrelated case, c.2024_2027delAGAG; p.(Glu675Glyfs*41), c.246-247delAG; p.(83Kfs*30), and c.457_461del; p.(Tyr153Serfs*5) were found in male patients with Encephalopathy with Status Epilepticus during Slow Sleep (ESES) [58]. They exhibited seizure, mild developmental delay, and language difficulties. More recently, c.1988_1989del; p(Arg663Asnfs*2), c.1653_1656del; p.(Asn551Lysfs*4), c.2005del; p.(Ala669Glnfs*48), and c.2026_2027del; p.(Arg676Aspfs*2) variants were found in four male patients with neurodevelopmental and epilepsy disorder [57]. Two patients had EEG documenting ESES and three of them were diagnosed as autism.

### 4.4. Splicing Variants of CNKSR2

Whole-exome sequencing showed a splicing variant of the *CNKSR2* gene (c.1904 + 1G > A) in a male with attention deficit, motor developmental delay, poor logical thinking ability, and introverted personality but without epilepsy [59]. This variant was predicted to cause a reading-frame shift and produced a truncated protein in the PH domain. In other unrelated cases, c.2145 + 1G > A, c.2044 + 2T > A, c.520 − 1G > A, and c.1905 − 2A > G in the *CNKSR2* were found in males [57]. Seizures, developmental delay, and language defects were reported in all of them and three of them were reported to have hyperactivity.

### 4.5. Cnksr2-Null Mouse Model

A *Cnksr2* knockout (KO) mouse line was recently developed [67]. The increased neural activity and spontaneous electrographic seizures were found in *Cnksr2* KO mice. In addition, these mice exhibited increased anxiety, defects in learning and memory, and a progressive loss of ultrasonic vocalizations. These phenotypes resembled symptoms of EAS patients and the KO mice may contribute to clarifying the pathophysiology of EAS.

## 5. Conclusions

To our knowledge, this is the first systematic review describing molecular characteristics and biological functions of CNKSR2. Additionally, we summarized the association between the gene variations of *CNKSR2* and neurodevelopmental disorders. The exact biological roles of CNKSR2 in vivo are largely unknown and further studies to clarify the functions of CNKSR2 in the brain may contribute to a better understanding of the pathophysiology of neurodevelopmental disorders.

## Figures and Tables

**Table 1:** Clinical features of patients with *CNKSR2* variations.

Family ID	Ethnicity	*CNKSR2* Variant ^a^	Gender	Affected Patients	Segregations	Intellectual Disability	Epilepsy/ Seizures	Hyper-Activity	Language Defect	Other Clinical Features	Ref.
#1	Norwegian	deletion Xp22.12 (21,375,312–21,609,484)	male	proband	maternal	mild/moderate	yes	yes	yes	borderline microcephaly	[8] [54]
#2	Canadian	deletion Xp22.12 (20,297,696–21,471,387)	male	proband	maternal	yes	yes	yes	yes		[54]
		deletion Xp22.12 (20,297,696–21,471,387)	male	brother	maternal	yes	yes	yes	yes		
		deletion Xp22.12 (20,297,696–21,471,387)	female	mother	NR	mild	no	NR	NR		
#3	French	deletion Xp22.12 (21,193,947–21,707,169)	male	proband	maternal	yes	yes	yes	yes	non-specific periventricular white matter hyperintensity	[54]
		deletion Xp22.12 (21,193,947–21,707,169)	male	brother	maternal	yes	no	yes	yes		
#4	French	frameshift (g.21,458,832_3insA, p.D152Rfs*8)	male	proband	maternal	yes	yes	yes	yes	minor cortical atrophy	[54]
		frameshift (g.21,458,832_3insA, p.D152Rfs*8)	male	brother	maternal	yes	febrile	yes	mild		
		frameshift (g.21,458,832_3insA, p.D152Rfs*8)	male	brother	maternal	yes	yes	yes	yes		
#5	NR	deletion Xp22.12 (21,328,677–21,670,497)	male	proband	maternal	yes	yes	NR	yes		[55]
#6	Ashkenazi	nonsense (c.2134 C > T, p.Arg712*) ^b^	male	proband	maternal	yes	yes	yes	yes		[53]
		nonsense (c.2134C > T, p.Arg712*) ^b^	male	brother	maternal	mild	yes	yes	mild		
		nonsense (c.2134C > T, p.Arg712*) ^b^	female	sister	maternal	mild	mild	NR	mild		
		nonsense (c.2134C > T, p.Arg712*) ^b^	female	mother	NR	no	febrile	NR	no		
#7	Chinese	nonsense (c.2185C > T, p.Arg729*)	male	proband	de novo	yes	yes	yes	yes	autism performance	[60]
#8	Dutch	nonsense (c.2304G > A, p.Trp768*)	female	proband	de novo	mild	yes	no	no		[61]
#9	Chinese	deletion Xp22.12 (21,606,698–21,616,207)	male	proband	maternal	yes	yes	yes	yes	white matter lesions	[56]
		deletion Xp22.12 (21,606,698–21,616,207)	male	brother	maternal	yes	yes	yes	yes	small multifocal white matter lesions	
		deletion Xp22.12 (21,606,698–21,616,207)	female	mother	de novo	mild	febrile	no	mild		
#10	Danish	frameshift c.2024_2027delAGAG, p.Glu675Glyfs*41	male	proband	de novo	mild	yes	NR	yes		[58]
#11	Spanish	frameshift c.246–247delAG, p.Thr83Lysfs*30	male	proband	de novo	mild	yes	yes	NR		[58]
#12	French	frameshift c.457_461del, p.Tyr153Serfs*5	male	proband	maternal	moderate/severe	yes	NR	yes		[58]
#13	French	deletion Xp22.12 (21,523,673–21,558,329)	female	proband	NR	mild	yes	yes	yes		[58]
#14	Spanish	deletion Xp22.12 (21,609,392–21,619,786)	male	proband	de novo	moderate	yes	yes	yes		[58]
#15	Chinese	splicing c.1904 + 1G > A	male	proband	maternal	mild	no	yes	no		[59]
#16	Chinese	nonsense c.625C > T, p.Gln209*	male	proband	maternal	yes	yes	yes	yes		[62]
#17	NR	nonsense c.2349T > G, p.Tyr783*	male	proband	de novo	yes	yes	yes	yes	autism	[57]
#18	NR	missense c.1537C > T, p.Pro513Ser)	male	proband	maternal	yes	yes	no	yes		[57]
#19	NR	frameshift c.1988_1989del, p.Arg663Asnfs*2	male	proband	de novo	yes	yes	yes	yes		[57]
#20	NR	frameshift c.1653_1656del, p.Asn551Lysfs*4	male	proband	de novo	yes	yes	no	yes		[57]
#21	NR	nonsense c.2545C > T, p.Arg849*	male	proband	maternal	yes	yes	yes	yes		[57]
#22	NR	splicing c.2145 + 1G >A	male	proband	de novo	yes	yes	yes	yes		[57]
#23	NR	deletion Xp22.12 (21,278,397–21,678,707)	male	proband	maternal	yes	yes	yes	yes	autism	[57]
#24	NR	nonsense c.1198C > T, p.Arg400*	male	proband	de novo	yes	yes	yes	yes		[57]
#25	NR	splicing c.2044 + 2 T>A	male	proband	de novo	yes	yes	yes	yes		[57]
#26	NR	splicing c.520 − 1G > A	male	proband	de novo	yes	yes	yes	yes		[57]
#27	NR	frameshift c.2005del, p.Ala669Glnfs*48	male	proband	de novo	yes	yes	no	yes		[57]
#28	NR	splicing c.1905 − 2A > G	male	proband	de novo	yes	yes	no	yes		[57]
#29	NR	frameshift c.2026_2027del, p.Arg676Aspfs*2	male	proband	de novo	yes	yes	yes	yes	autism	[57]

^a^ Human genome version 19 by UCSC genome browser and the human *CNKSR2* (NM_014927). ^b^ The variant was described as ’c.2314 C > T, p.Arg712*’ in the literature; but the amino acid position of Arg712 is relevant to the nucleic acid position of 2134_2136. The number is counted from the translational start site. NR: not reported.
